# Biological Thermal Performance of Organic and Inorganic Aerogels as Patches for Photothermal Therapy

**DOI:** 10.3390/gels8080485

**Published:** 2022-08-03

**Authors:** Tânia Ferreira-Gonçalves, Ana Iglesias-Mejuto, Teresa Linhares, João M. P. Coelho, Pedro Vieira, Pedro Faísca, José Catarino, Pedro Pinto, David Ferreira, Hugo A. Ferreira, Maria Manuela Gaspar, Luísa Durães, Carlos A. García-González, Catarina Pinto Reis

**Affiliations:** 1 Research Institute for Medicines (iMed.ULisboa), Faculty of Pharmacy, Universidade de Lisboa, Av. Professor Gama Pinto, 1649-003 Lisboa, Portugal; taniag1@edu.ulisboa.pt (T.F.-G.); pfcpinto@ff.ulisboa.pt (P.P.); mgaspar@ff.ulisboa.pt (M.M.G.); 2 Instituto de Biofísica e Engenharia Biomédica, Faculdade de Ciências, Universidade de Lisboa, Campo Grande, 1749-016 Lisboa, Portugal; jmcoelho@fc.ul.pt (J.M.P.C.); hugoferreira@campus.ul.pt (H.A.F.); 3 I+D Farma Group (GI-1645), Department of Pharmacology, Pharmacy and Pharmaceutical Technology, Faculty of Pharmacy, iMATUS and Health Research Institute of Santiago de Compostela (IDIS), Universidade de Santiago de Compostela, E-15782 Santiago de Compostela, Spain; ana.iglesias.mejuto@rai.usc.es (A.I.-M.); carlos.garcia@usc.es (C.A.G.-G.); 4 University of Coimbra, CIEPQPF, Department of Chemical Engineering, 3030-790 Coimbra, Portugal; tcalinhares@gmail.com (T.L.); luisa@eq.uc.pt (L.D.); 5 2C2T-Centre for Textile Science and Technology, University of Minho, Campus of Azurém, 4800-058 Guimarães, Portugal; 6 Physics Department, NOVA School of Science and Technology (Campus de Caparica), 2829-516 Caparica, Portugal; pmv@fct.unl.pt; 7 CBIOS-Research Center for Biosciences & Health Technologies, Universidade Lusófona de Humanidades e Tecnologias, Campo Grande 376, 1749-024 Lisboa, Portugal; pedrofaisca@ulusofona.pt; 8 Faculdade de Medicina Veterinária, Universidade Lusófona de Humanidades e Tecnologias, Campo Grande 376, 1749-024 Lisboa, Portugal; p5663@ulusofona.pt; 9 Comprehensive Health Research Centre (CHRC), Departamento de Desporto e Saúde, Escola de Saúde e Desenvolvimento Humano, Universidade de Évora, Largo dos Colegiais, 7004-516 Évora, Portugal; david.ferreira@uevora.pt

**Keywords:** pectin aerogels, silica aerogels, light delimiters

## Abstract

Aerogels are materials with unique properties, among which are low density and thermal conductivity. They are also known for their exquisite biocompatibility and biodegradability. All these features make them attractive for biomedical applications, such as their potential use in photothermal therapy (PTT). This technique is, yet, still associated with undesirable effects on surrounding tissues which emphasizes the need to minimize the exposure of healthy regions. One way to do so relies on the use of materials able to block the radiation and the heat generated. Aerogels might be potentially useful for this purpose by acting as insulators. Silica- and pectin-based aerogels are reported as the best inorganic and organic thermal insulators, respectively; thus, the aim of this work relies on assessing the possibility of using these materials as light and thermal insulators and delimiters for PTT. Silica- and pectin-based aerogels were prepared and fully characterized. The thermal protection efficacy of the aerogels when irradiated with a near-infrared laser was assessed using phantoms and ex vivo grafts. Lastly, safety was assessed in human volunteers. Both types presented good textural properties and safe profiles. Moreover, thermal activation unveils the better performance of silica-based aerogels, confirming the potential of this material for PTT.

## 1. Introduction

Aerogels are a group of porous materials that have been attracting attention from the scientific community [1,2,3,4] since their first report in the 1930s by Kistler [5]. Aerogels have been defined as highly porous materials whose pores’ filling phase is a gas, possibly obtained from gels through different drying processes that must not compromise the gel structure significantly [6]. However, there are other definitions found in the literature [4,7]. Generally, aerogels present special characteristics, namely very low density (<0.5 g·cm^−3^), high surface area, high porosity (>80%) with controllable pore size in the nanoscale, tunable surface chemistry, and physical properties, and low thermal conductivity, refractive index and dielectric constant [2,3,4,8]. In addition, aerogels may also present great biodegradability, biocompatibility, permeability, and ability to mimic biological structures [1,2,8], making them ideal candidates for biomedical and pharmaceutical applications [9].

Multiple aerogels made of different materials were already reported and associated with distinctive features [2,8]. Depending on the nature of the materials, aerogels can be divided into inorganic, organic, and hybrid. Inorganic aerogels include metals—(e.g., Au, Pt, Ag, etc.), oxide—(TiO_2_, Al_2_O_3_, Fe_2_O_3_, etc.), silica—(only SiO_2_ or hybrid SiO_2_), and chalcogen-based (CdS, ZnS, PbTe, etc.) aerogels [2,8]. In its turn, organic aerogels include carbon-based (e.g., nanotubes, graphene, etc.) and polymer-based aerogels [1,2,8,10], among which there are bio-based aerogels. Lastly, hybrid aerogels combine in the same structure organic and inorganic materials [11,12,13]. Amongst all aerogels, silica-based aerogels are the most explored and common [2,10,14,15], being associated with high porosity, transparency, and one of the lowest thermal conductivities, which makes them great thermal insulators. However, this type of aerogel is typically brittle and presents low resistance to compression [10,14]. Nevertheless, several strategies relying on silica reinforcement with other materials and fibers, such as cotton fibers, have been proposed and allowed to obtain aerogel structures with similar textural properties but with fewer fragility problems [12,16,17,18,19]. Furthermore, alternative materials with interesting thermal insulating properties and increased resistance have been explored. In this context, biopolymer-based aerogels have attracted attention since some biopolymers showed promising thermal insulating properties while simultaneously representing more biocompatible, biodegradable, resistant, and environmental-friendly alternatives [8,10,14]. Among biopolymer-based aerogels, pectin-based aerogels are reported as one of the best thermal insulating materials [10,14,20]. Apart from the material, aerogels’ properties also depend on the preparation method used. The preparation of aerogels generally starts with sol-gel chemistry, followed by the replacement of the solvent with a gas using proper drying methodologies that shall not significantly reduce the volume or compromise the 3D-network structure [2,8]. Intermediate steps can be used, such as aging (to increase the network and cohesion), washing of by-products, or solvent exchange. The final step, the drying, can be carried out, for instance, by ambient pressure drying, freeze-drying, or supercritical drying [4,8]. Supercritical drying relies on subjecting the structures to a certain temperature and pressure conditions, such as the liquid filling the pores reaching its supercritical conditions, then becoming impossible to distinguish the liquid and vapor states, minimizing the capillary forces on the pores and avoiding the pores collapse and material shrinkage [2,8,21]. This process may require a solvent exchange step to change the solvent of the gel for a suitable solvent to be supercritically extracted, such as ethanol. This methodology for aerogel preparation avoids the need for post-processing steps and surpasses the main challenges associated with the other drying methodologies, namely the microstructures collapse and major volume shrinkage [4,8,21].

Several biomedical applications of aerogel structures have already been proposed, including drug delivery, biosensing, implementable devices, regenerative medicine, tissue engineering, and diagnostic tools [1,2,8,22,23,24]. Aerogels have also been proposed for promising therapeutic strategies such as photothermal therapy (PTT) [25,26,27]. PTT appeared as an increasingly explored therapeutic option for superficial localized tumors relying on the induction of local hyperthermia of tumor cells upon irradiation with light beams [28,29]. It represents a tumor-preferred effect since cancer cells are less capable of dissipating heat than healthy cells [30], being, consequently, more sensitive to it. Moreover, PTT is less invasive and presents faster recovery periods when compared with standard oncology treatments, such as chemotherapy [31]. However, the value of PTT depends greatly on the ability of radiation to penetrate to a certain depth in the tissues and on the ability to generate enough heat able to damage tumor cells. One option to surpass those limitations might include using, in the same system, near-infrared (NIR) radiation and photothermal enhancers such as gold nanoparticles [30,31,32,33] or hybrid aerogels [25,26,27]. NIR radiation represents an advantageous practice because it is less absorbed and scattered by the tissues [29,34], leading to enhanced penetration depth. Despite the numerous improvements made in PTT systems, these therapies are still associated with undesirable effects of the light on surrounding healthy tissues [35]. This drawback could be surpassed by delimiting and blocking the radiation beam. Typical light delimitation systems could be implemented, but they include complex optical systems [36,37], and thermal effects outside the area of interest still have to be considered. For this intent, light and thermal insulators might be promising materials, and aerogels are known for their excellent thermal insulating features.

The aim of this work was to assess the value of pectin- and silica-based aerogels as light and heat delimiters and insulators in PTT systems. Our group had previously proposed the use of PTT as a promising therapeutic strategy for breast and thyroid cancers and for melanoma [38,39,40]. The results obtained were very encouraging, yet there is still plenty of room for improving the proposed system. Despite no skin burns or damage have been reported in any of the published works from our group, the current irradiation system lacks the ability to control the laser beam diameter, which hinders the tunning of the irradiation area according to the experimental model used. Thus, herein it is proposed the use of aerogels as light and thermal delimiters and insulators in a PTT system, an application of aerogels not yet reported elsewhere to the best of the authors’ knowledge.

## 2. Results and Discussion

Organic and inorganic aerogels made of pectin and silica reinforced with cotton fibers were prepared by ethanol- and thermal-mediated gelation with supercritical-drying and a two-step catalyzed sol-gel method followed by oven drying, respectively, and were physiochemically characterized. The biological and thermal performance of aerogels when combined with irradiation with a NIR laser was assessed using agar phantoms and pig and human ex vivo skin grafts as experimental models. Temperature increments caused by laser radiation exposure of the experimental models, as well as morphological alterations, were analyzed. The safety of both aerogels was also assessed to rule out their value as light and thermal delimiters and insulators to be applied as patches in a PTT system.

### 2.1. Physicochemical Characterization

**Figure 1:**
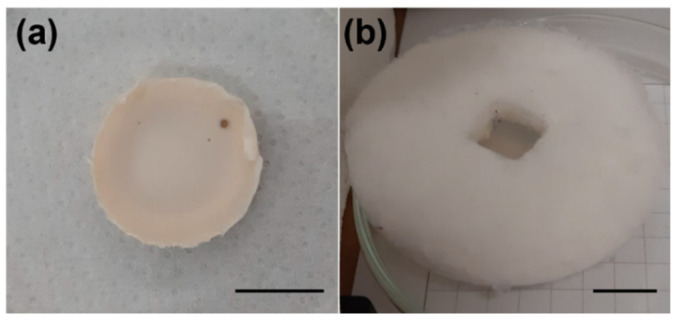
Macroscopic aspect of the prepared aerogels: (**a**) pectin-based, (**b**) silica-based.

Pectin- and silica-based aerogels were successfully prepared following protocols previously reported in the literature [12,21]. Representative pictures of the obtained structures are presented in Figure 1. There are clear macroscopic differences between the two types of structures. Pectin-based aerogels were very rigid and presented smooth surfaces. Moreover, these structures showed some surface irregularities/deformations that appeared during the process of solvent exchange. In contrast, silica-based aerogels were soft but easily flaky. In addition, silica-based aerogels presented very regular surfaces. Silica-based aerogel presents a central hole with about 1 cm^2^, which was pre-made on purpose for posterior tests with a laser.

**Figure 2:**
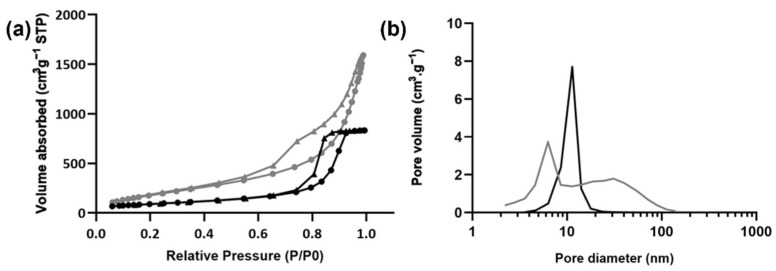
BJH analysis of pectin-based (black) and silica-based (grey) aerogels. (**a**) Adsorption (circles)/desorption (triangles) isotherms and (**b**) specific pore volume distribution.

Physicochemical and textural properties of aerogels were assessed (Table 1). A very high volume shrinkage was observed for pectin-based aerogels in contrast to what was observed for silica-based aerogels. The volume shrinkage verified for pectin structures was much higher than what is typically described in the literature for similar structures (about 48 ± 28% [21]), unveiling poor textural properties of the structures herein prepared. These suspicions were also supported by the bulk density (*ρ_bulk_*) observed, which was about two times higher than what was reported before (<0.15 g·cm^−3^) [14,20,21]. Nevertheless, textural analysis of pectin-based structures showed high specific surface area (*A_BET_*) and reduced specific pore volume (*Vp_,BJH_*) and pore diameter (*dp_,BJH_*). In fact, the A_BET_ observed was even slightly higher, and the pore diameter slightly smaller than what was previously published by others [21]. The volume and diameter of the pores herein reported were assessed by BJH approach, an analytical method widely used yet presenting some limitations that require a certain caution in the analysis of the results [41]. BJH-method typically underestimates the actual pore diameter from the aerogel samples, as it only considers pores within the range of 1.7–300 nm and neglects macropores visible in SEM analysis [20,41]. Accordingly, *dp_,BJH_* values may be smaller than the actual mean pore size values for these aerogels. In addition, the overall properties of pectin-based aerogels depend on the type and concentration of pectin, the gelation method, and also on the drying technique [14,20,42,43], which explain the differences observed in comparison with previously published data. In its turn, silica-based aerogels presented low bulk densities in agreement with what was reported in the literature [12]. Silica-based aerogels presented very high *A_BET_* values, more than double of pectin-based structures. Similar pore diameters were observed for the two types of aerogels, but silica-based aerogels presented higher pore volumes. The isotherms and pore size distribution resultant from low-temperature N_2_ adsorption/desorption analysis are presented in Figure 2. Both aerogels’ isotherms are from type IV (hysteresis), according to the IUPAC classification [44], which is typical of mesoporous materials. Altogether, the textural properties from both aerogels unveil promising results in terms of thermal insulating features.

**Figure 3:**
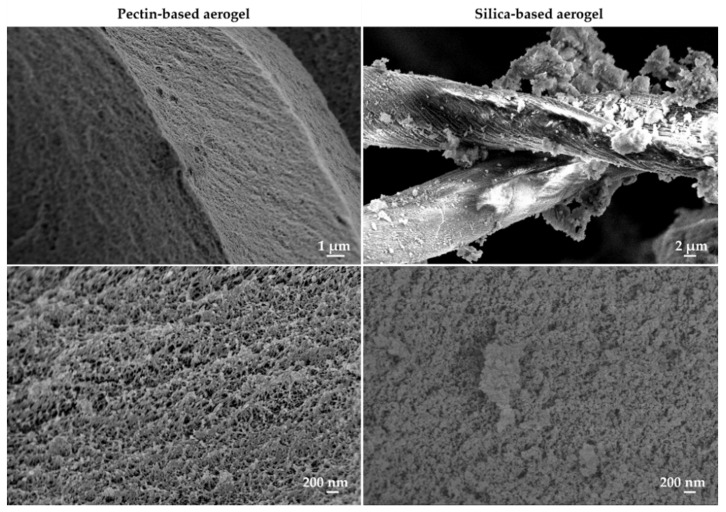
Scanning electron microscopy (SEM) images of pectin-based (**left** images) and silica-based (**right** images) aerogels at different magnifications.

The morphology of aerogels was analyzed by scanning electron microscopy (SEM) (Figure 3), where it is possible to see different-sized pores, namely mesopores and macropores. This observation refers both to pectin-based and silica-based aerogels. Moreover, it is possible to clearly identify the presence of the cotton fibers in the silica-based aerogel.

The portion of an incident light that passes through aerogels, also known as transmittance, was determined upon irradiation of the structures with a NIR laser (808 nm). Subsequently, attending the Beer-Lambert law, the attenuation coefficient was determined for each one of the aerogels for a known thickness. Pectin-based aerogels presented an attenuation coefficient of 9.3 ± 1.2 cm^−1^ in comparison with the 4.6 ± 0.1 cm^−1^ observed for silica-based aerogels. The high standard deviation observed for the pectin-based structures might be a consequence of their surface irregularities that may result in different reflection, absorbance, and scattering phenomena. These results showed that pectin-based aerogels are able to absorb more NIR radiation per unit of area than silica-based aerogels, which possibly means that they are also more prone to irradiation-induced heating than silica-based aerogels.

**Figure 4:**
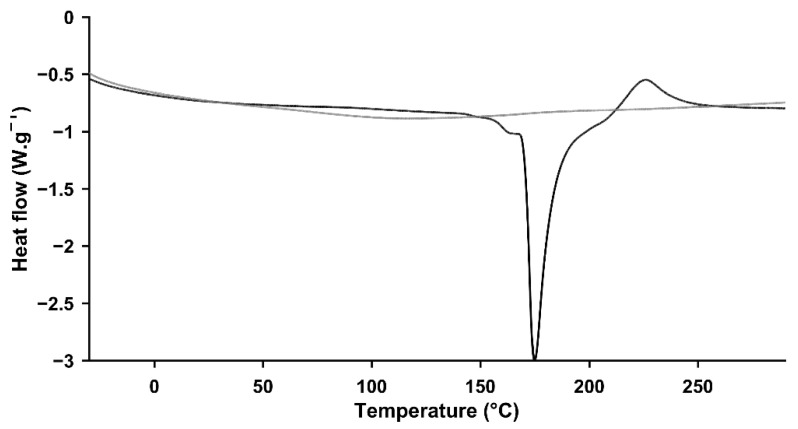
Modulated differential scanning calorimetry (mDSC) thermograms of pectin- (black line) and silica-based aerogels (grey line).

Modulated differential scanning calorimetry (mDSC) thermograms of pectin-based aerogels (Figure 4) had an endothermic event at approximately 172.2 ± 1.9 °C (with an enthalpy of 304.8 ± 3.5 J·g^−1^), followed by an exothermic event at 226.0 ± 0.5 °C. As described previously [45], pectin presents melting temperatures that can range from 120 up to 180 °C, in line with the findings herein obtained. Concomitantly, the observed exothermic event at 226.0 °C is possibly justified based on the degradation of the polysaccharide at temperatures above 220 °C [10,14]. In contrast, thermograms of silica-based aerogels failed to present endothermic or exothermic events during the temperature range considered. Consequently, silica-based aerogels present higher thermal stability compared to those samples prepared based on pectin.

### 2.2. Biological Performance of Aerogels When Combined with NIR Irradiation

**Figure 5:**
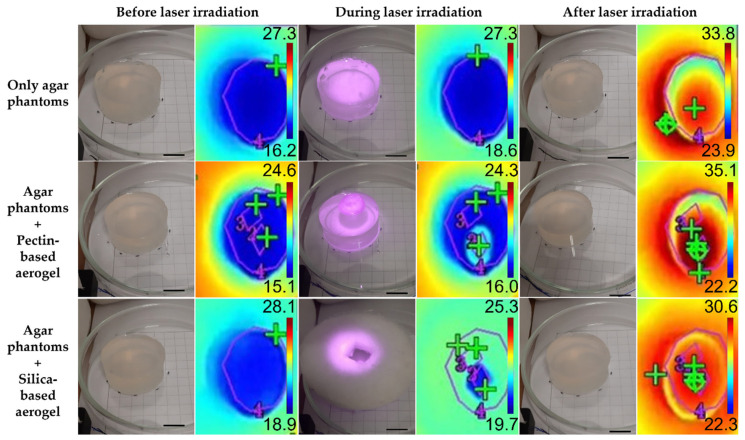
Real and thermal images of agar phantoms before, during (after 1 min of irradiation) and after laser irradiation. Scale bar: 1.3 cm.

**Figure 6:**
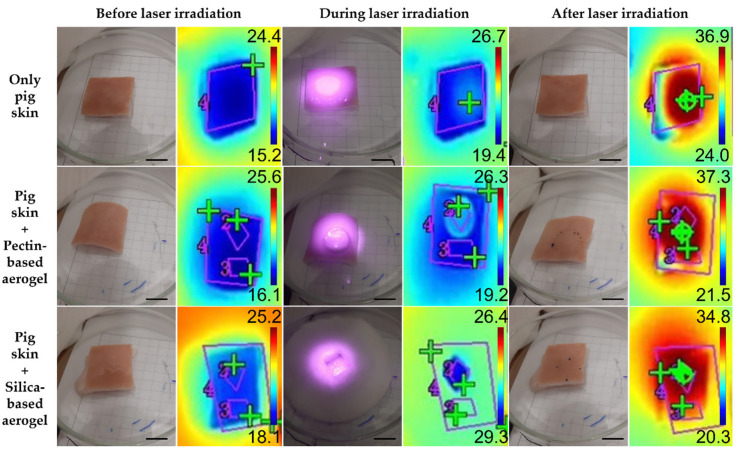
Real and thermal images of pig skin ex vivo grafts before, during (after 1 min of irradiation) and after laser irradiation. Scale bar: 1.3 cm.

**Figure 7:**
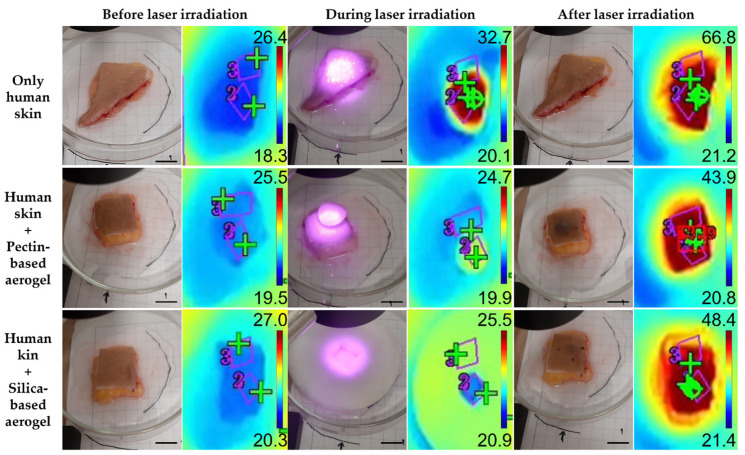
Real and thermal images of human skin ex vivo grafts before, during (after 1 min of irradiation) and after laser irradiation. Scale bar: 1.3 cm.

The biological performance of aerogels was assessed using three experimental models: agar phantoms (Figure 5), pig skin ex vivo grafts (Figure 6), and human skin ex vivo grafts (Figure 7). The irradiation, kept for 15 min, was carried out directly on top of the experimental models or on top of the aerogel structures, which, in turn, were placed directly on top of the models. For comparison of the temperature increment over irradiation time within the same model in a test group using aerogels, two areas were distinguishable: (a) irradiated area—corresponding to an area directly irradiated; (b) non-irradiated area—corresponding to the aerogel temperature during irradiation and to the temperature of the area under the aerogel once the laser was turned off and removed from the model.

**Figure 8:**
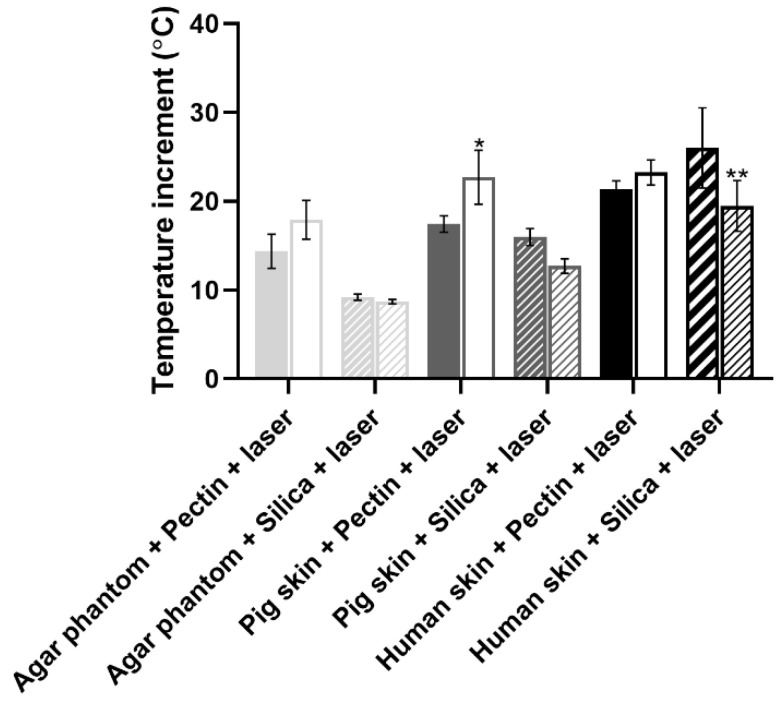
Temperature increment observed for agar phantoms and pig and human skin ex vivo grafts after 15 min of NIR laser exposure. For each test group, full columns (left column) refer to directly irradiated areas, whereas empty and stripped columns (right) refer to areas under aerogel structures being irradiated. Statistical significance represents * *p* < 0.05 and ** *p* < 0.01 compared with directly irradiated areas from the same group.

In order to present a more complete analysis, the temperature increment was analyzed over irradiation time with the laser turned ON (Figures S1–S3 for the agar phantoms, pig skin grafts, and human skin grafts, respectively) and attending only to the final temperature once the laser was turned OFF, a more controlled situation with no energy supply involved (Figure 8). The temperature increments over time were analyzed with a certain caution because the thermal signal was being detected throughout the irradiation process having been impossible to isolate the interference from the light of the laser reflected on any of the materials interacting with the laser beam. The thermal camera relies on the detection of radiation within the far NIR wavelength range, whereas the wavelength from the laser belongs to the low NIR; thus, it is expected that the interference of the laser signal is neglectable. Nonetheless, to simplify the measurements, temperature increments were analyzed rather than absolute temperatures, as absolute temperature measurements would imply a prior calibration of the thermal camera according to the emissivity of each one of the materials. Overall, regardless of the experimental model, the pectin-based aerogel (not the area under the aerogel) reached the highest temperatures, presenting temperature increments higher than 40 °C. In contrast, silica-based aerogels heated up the least, less than 10 °C, heating even less than the models being directly irradiated in the case of both pig and human skin grafts. Attending directly irradiated areas, no significant differences (*p* < 0.05) were seen between areas directly irradiated in the presence or absence of any of the aerogel’s structures. Exceptions were in the case of agar phantoms, in which areas directly irradiated when the pectin-based aerogel was being irradiated heated up more than areas directly irradiated without the presence of an aerogel or in the presence of an aerogel of silica. Even though this phenomenon is more notorious in agar phantoms, pig skin grafts also present similar behavior. It is hypothesized that it can be a consequence of cumulative effect, in which the temperature increment observed can result from the natural warming caused by the direct irradiation in combination with the warming caused by heat dissipation from the pectin-based structure.

Attending the overall temperature increment of the models in areas directly irradiated and areas under the aerogel structure, small differences were seen (Figure 8). Regardless of the experimental model, areas under pectin-based aerogels after 15 min of irradiation always presented slightly higher temperatures than the directly irradiated counterparts, even though the differences were not statistically significant (*p* < 0.05), except in pig skin grafts. This higher temperature increase might be a consequence of a cumulative effect of direct irradiation with heat dissipation from the pectin structure. In contrast to what was seen for the pectin-based aerogels, areas under silica-based aerogels presented slightly smaller temperature increases than directly irradiated areas in all the experimental models used. These results point out that silica-based aerogels seem to be more promising light and thermal insulators for biomedical applications using NIR radiation. At last, it is clear that the amplitude of the temperature increment observed depended on the experimental model. This might happen due to different interactions of the light (absorbance, scattering, and reflection) with the different models, as well as due to different heat dissipation features [46]. Agar phantoms are transparent and thus present lower optical absorption and scattering than the skin [46]. The differences reported between the different experimental models herein selected are, however, advantageous for assessing the value and efficacy of the system in different scenarios. These differences mimic what happens in real life as the skin of each person responds differently to light exposure depending on factors such as hair presence, skin color, hydration level, fat content, and prior skin lesions, among others [34].

**Figure 9:**
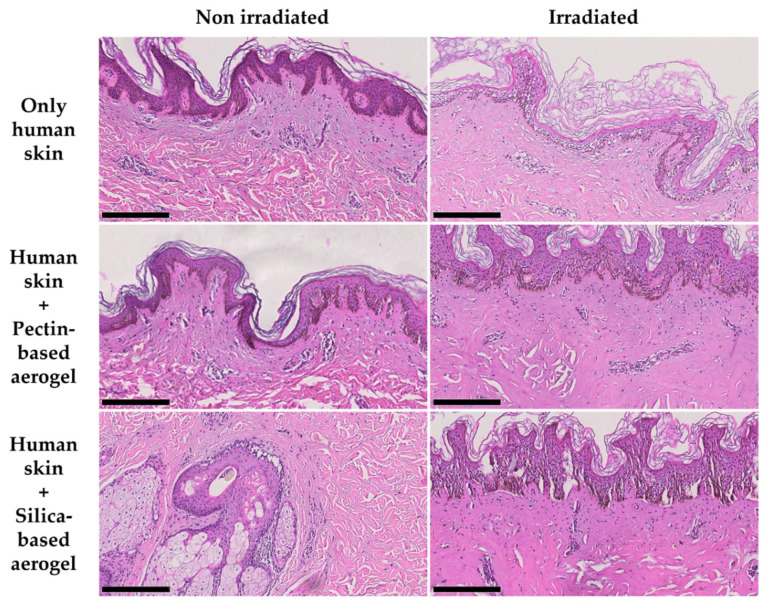
Histological images (H&E staining) of human skin ex vivo grafts subjected or not to laser irradiation directly or combined with a pectin-based or silica-based aerogel structure. All images are representative of each test group. Scale bar: 250 µm.

After irradiation, skin grafts were subjected to histopathological analysis to assess if there were any differences between test groups that were not possible to observe macroscopically. In the case of pig skin grafts, no differences were observed between any of the test groups as all the skin excerpts showed the same skin alterations possibly resultant from the singe. Despite these data supporting the macroscopical observations, further tests must be carried out. In its turn, analysis of the human skin grafts showed some differences between testing groups (Figure 9 and Table 2). Firstly, both aerogels proved to be completely safe for the human skin after direct contact for 15 min, as no morphological alterations were observed when compared with control skin (not subjected to any treatment). Direct irradiation of the skin without any aerogel led to hydropic degeneration of the keratinocytes from the epidermis, a reversible alteration [47] also seen in the skin portions under both aerogel structures after irradiation, although the areas under the aerogels presented milder alterations (Table 2). In its turn, portions of skin directly irradiated from areas surrounding the aerogels, in addition to hydropic degeneration of keratinocytes from the epidermis, also showed coagulative necrosis from the dermis, with the morphological alteration being more notorious in the skin irradiated in the presence of pectin-based aerogels. All this evidence supports the hypothesis of the cumulative effect of light and heat dissipation.

### 2.3. Preliminary In Vivo Safety Assays Using Artemia salina Model

**Figure 10:**
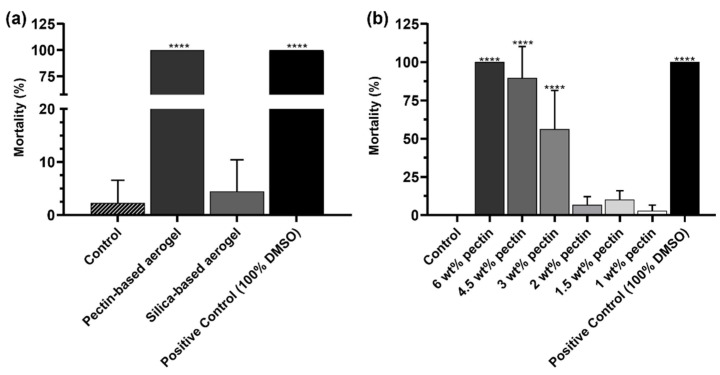
Mortality (%) of *Artemia salina* incubated for 24 h with aerogel structures (**a**) and with pectin solutions at different concentrations (**b**). *Artemia* salt medium (Control) was used as negative control of mortality (%), whereas 100% of DMSO was used as positive control. The results represent the mean value ± SD, *n* > 4. Statistical significance represents **** *p* < 0.0001 compared with the group incubated only with *artemia* salt medium.

The toxicity of the aerogel structures was assessed in *Artemia salina*, a simple preliminary in vivo model. As shown in Figure 10a, silica-based aerogels did not present toxicity. On the opposite, pectin-based aerogels presented very high toxicity to *Artemia salina*. It was hypothesized that the reason behind the toxicity could be the pectin concentration. Thus, further tests were carried out with pectin solutions at different concentrations. *Artemia salina* toxicity assay revealed the concentration dependance of the mortality (%) (Figure 10b). Pectin concentrations higher than 3 wt. % showed high toxicity, whereas lower concentrations were revealed to be completely safe in the same experimental model. The concentration-dependence toxicity herein observed had also been reported in cellular models [48,49].

### 2.4. Human Skin Compatibility Tests

Results from human skin compatibility tests showed no reaction in none of the volunteers after 48 ± 5 h of exposure, regardless the aerogel material (Table S1). These results confirmed a very good compatibility and safety of both pectin-based and silica-based aerogels for topical applications.

## 3. Conclusions

Two different types of aerogels were successfully prepared and characterized to further assess their possible use as light and thermal delimiters and insulators in biomedical applications using non-ionizing radiation as therapy. The pectin-based aerogels were prepared using as the main constituent a polysaccharide with biological origin easily accessible, representing a more environmentally friendly alternative than silica-based aerogels. Pectin-based aerogels presented promising textural properties despite the high volume shrinkage, and density reported when compared with previous values reported by others, which was very encouraging for the use of these structures as thermal and light insulators. In its turn, silica-based aerogels presented exquisite textural properties in similarity to the reported in the literature and are associated with great thermal insulating properties. Moreover, silica-based aerogels were softer than pectin-based aerogels. Attending to the thermal tests, pectin-based aerogels did not prove to have the expected thermal, and light insulators in the conditions herein applied. Contrarily, silica-based aerogels were revealed to be promising thermal insulators. Attending to the biological safety, contrarily to pectin-based aerogels, silica-based aerogels proved to be safe in an *Artemia salina* model, having been noticed that the toxicity of pectin in this model was concentration-dependent. Nevertheless, when the safety of the same structures was evaluated in skin models both ex vivo in pig and human skin grafts and in vivo in human volunteers, none of the materials showed any skin irritation or other alteration. Thus, overall, silica-based aerogels proved to be better thermal and light insulators for therapeutic alternatives using non-ionizing radiation.

## 4. Materials and Methods

### 4.1. Materials

Pectin from apple (76282, degree of esterification—DE: 70–75%), citrus peel (P9135) and methyltrimethoxysilane (MTMS, 98%) were supplied from Sigma-Aldrich (Steinheim, Germany). Hexamethyldisilazane (HMDZ, 98.5%) from abcr GmbH (Karlsruhe, Germany), and ammonium hydroxide (NH_4_OH, puriss. p.a.) from PanReac AppliChem (ITW Reagents, Barcelona, Spain). Tetraethyl orthosilicate (TEOS, 98%) was bought from Acros Organics and Ethyl acetate (EtOAc ≥ 99.8%) and acetic acid (CH_3_COOH, glacial, analytical reagent grade) were supplied from Fisher Chemical and Fisher Scientific, all belonging to the Thermo Fisher Scientific group (Waltham, MA, USA). Ethanol absolute was purchased from VWR (Radnor, PA, USA). The water used was purified through a Millipore system. CO_2_ (purity > 99%) was supplied by Nippon Gases (Madrid, Spain). Commercial hydrophilic cotton fibers were used as the reinforcement matrix, previously aligned in the lengthwise direction. All the remaining chemicals used were of analytical grade.

### 4.2. Preparation of Pectin-Based Structures

#### 4.2.1. Pectin Hydrogel Preparation

Pectin-based structures were prepared in accordance with a method previously reported by García-González et al. [21], which combined both ethanol addition and temperature for the gelation process. The addition of ethanol increases the viscosity of the solutions by promoting pectin-pectin interactions rather than pectin-solvent interactions [21]. Initially, an aqueous solution with 6 wt. % of a mixture of both citrus peel and apple pectin (1:1, *w*/*w*) was prepared using mechanical stirring (500 rpm and room temperature) (VWR VOS 60, Radnor, PA, USA) until complete homogenization. As a note, the mass of pectin to use was calculated based on Equation (1). Thereafter, the solution was heated up at 50 °C (RCT basic IKAMAG^®^ safety control, IKA^®^-Werke GmbH & Co. KG, Staufen, Germany) under mechanical agitation (500 rpm) for 30 min. Then, 15 wt. % of ethanol was added to the solution (weight calculated according to Equation (2)), and it was kept stirring at 50 °C for 5 min more. At this point, the gel was ready to be transferred to the molds, consisting of 6-well plates.

(1) $$Pectin\;\mathrm{wt}.\;\%=\frac{m_{Pectin}}{m_{Pectin}+m_{water}}\times 100$$

(2) $$Ethanol\;\mathrm{wt}.\;\%=\frac{m_{ethanol}}{m_{ethanol}+m_{water}}\times 100$$

#### 4.2.2. Supercritical Drying

Pectin-based aerogels were obtained through supercritical drying, which required a prior solvent exchange step to switch water to ethanol. The solvent exchange was carried out in two equal subsequent steps by complete immersion of the structures in ethanol 100%, in which every step was carried out for a minimum of 24 h. After solvent exchange, the pectin-based structures were dried by supercritical CO_2_. The alcogels were firstly loaded in paper envelops and then placed in an autoclave (Thar Process, Pittsburg, PA, USA) filled with pure ethanol until covering all the structures. The autoclave temperature was increased up to 40 °C, and a working pressure of 120–130 bar was reached. A continuous flow of 6 g·min^−1^ of supercritical CO_2_ was applied, and the samples were dried for ≈ 4 h. At last, the pressure on the autoclave was slowly decreased (controlled depressurization) until the atmospheric pressure was reached. This methodology was previously reported [22,23,50].

### 4.3. Preparation of Silica-Based Structures

Silica-based structures were prepared based on a two-step catalyzed sol-gel method previously described by Linhares et al. [12]. TEOS and MTMS, precursor and co-precursor, respectively, were firstly mixed in a volume ratio of 4:1 and hydrolyzed for 24 h at 27 °C, in a solution of ethanol, water, and acetic acid. Afterward, ammonium hydroxide (1 M) was added to the solution to boost the condensation/gelation. The followed molar ratio was Si (TEOS/MTMS):EtOH:H_2_O:CH_3_COOH:NH_4_OH = 1 (0.72/0.28):6.85:8.67:3.69 × 10^−2^: 7.00 × 10^−2^. Afterward, about 14 wt. % of cotton fibers were disposed layer-by-layer, and the hydrolyzed solution was poured over it. The gels were then aged at 50 °C in an oven (MOV-212S, SANYO Electric Co. Ltd., Osaka, Japan) for 3 days. For the aging, a certain volume of ethanol was added to cover the gels (up to 3 mm from the gels surface) and the gel container was properly sealed to guarantee that it was airtight. Subsequentially, the gels were hydrophobized with a solution of HDMZ in EtOAc (20% vol., pre-prepared at 55 °C for 1 h) at 50 °C for 48 h. Lastly, the gels were washed twice with EtOAc at 50 °C during 5 h and dried at 150 °C for 2 h.

### 4.4. Physicochemical Characterization of the Aerogels

The size of the gel structures was measured using an electronic digital caliper (Fowler & NSK MAX-CAL, Fowler High Precision, Newton, MA, USA). The volume shrinkage % of the structures was calculated as follows:

(3) $$Shrinkage\;\%=\left(\frac{V_{i}-V_{f}}{V_{i}}\right)\times 100$$

with *V_f_* representing the volume after all the synthetic procedure and *V_i_* the initial volume of the structures before drying. The volume was calculated after measuring the aerogels diameter (d) and height (h) assuming a cylindrical shape.

The bulk density (*ρ_bulk_*) of the structures was determined by measuring the dimensions and weighting the samples, using the following equation:

(4) $$\rho_{bulk}=\frac{m_{aerogel}}{V_{f}}$$

with *m_aerogel_*, the weight of the sample.

The textural properties of aerogels were assessed by Nitrogen adsorption-desorption measurements (ASAP 2000 Micromeritics Inc, Norcross, GA, USA) after drying the samples for more than 20 h under vacuum at 800 µm·Hg and 60 °C. The specific surface area (*A_BET_*) was determined by the Brunauer-Emmett-Teller (BET) method, whereas the specific pore volume (*V_p,BJH_*) and the mean pore diameter (*d_p,BJH_*) were estimated using the Barrett-Joyner-Halenda (BJH) method. BJH method was used on the desorption branch of the isotherm and measured pores in the range of 1.7 to 300 nm.

Aerogels’ morphology was characterized by scanning electron microscopy (SEM, EVO LS15, Zeiss, Oberkochen, Germany) after sputtering iridium onto the structures to minimize charging effects and enhance the quality of the acquired images.

The absorption coefficient of aerogels for the wavelength used in the irradiation tests was obtained using the methodology detailed by Merillas et al. [51]. The samples were placed in the 11.5 mm diameter window of an integrating sphere (2P4/M Integrating Sphere, ThorLabs, Newton, NJ, USA) and irradiated by a 6 mm diameter collimated beam emitted by a NIR diode laser (JDSU L4-2495-003 Diode Laser, JDSU, Milpitas, CA, USA coupled to a LaserPak laser diode driver ARO-485-08-05, Arroyo Instruments, LCC, San Luis Obisco, CA, USA). The signal was measured by a photometer (PDA200C, Thorlabs, Newton, NJ, USA) attached to the integrating sphere. The attenuation coefficient of the samples (*µ*) was determined according to the Beer-Lambert equation [52]:

(5) $$\mu =-\frac{\ln T}{x}$$

where *x* is the samples’ thickness, and

(6) $$T=\frac{I}{I_{0}}$$

with *T* the transmittance of the sample, and *I*_0_ and *I* the incident and transmitted irradiances, respectively.

Modulated differential scanning calorimetry (mDSC) was considered to evaluate the thermal behavior of samples in a TA calorimeter (DSC Q200, TA Instruments, New Castle, DE, USA), coupled to a DSC refrigerated cooling system (TA Instruments, New Castle, DE, USA). Samples (*n* = 2, 5.0–10.8 mg) were hermetically sealed inside aluminum pans before analysis. Indium, as reference, was used to calibrate the equipment (temperature and enthalpy). A modulated analysis between −40 and 300 °C using a heating rate of 5 °C·min^−1^ and an amplitude of 0.796 °C during 60 s was applied at a constant nitrogen gas flow (50 mL·min^−1^). Data analysis was performed using proprietary software (Universal Analysis 2000, version 4.7A, 2009, TA Instruments, New Castle, DE, USA).

### 4.5. Biological Performance of Aerogels When Combined with NIR Irradiation

The biological thermal and light insulation properties of the aerogels were assessed using three different experimental models: agar phantoms, ex vivo pig skin grafts and ex vivo human skin grafts. Agar phantoms were prepared by aqueous dissolution of 1% (w/v) of agar under manual agitation and heating, followed by the transfer of the solution into 6-well plates posteriorly stored at 2 °C until complete gelation. Once agar was completely geled, the structures were detached from the plate wells, being obtained transparent cylindrical structures. Ex vivo pig skin grafts consisted in approximately squared pieces (≈2.5 cm × 2.5 cm, with thicknesses around 0.5 cm) of skin from the pig loin bought at a local butchery. When bought, the pig skin did not present any hair and only had a thin layer of fat tissue (<0.5 cm). Moreover, as soon as the skin reached the lab it was kept in phosphate-buffered saline (PBS 1x, pH 7.4, USP32). Ex vivo human skin grafts were prepared from a piece of skin collected *postmortem* from the belly region of a female patient kindly donated by a medical center. Immediately after excision, the skin was immersed into PBS 1x and kept in refrigerated conditions for transportation.

Once in the laboratory facilities, the excess of fat was excised with a scalpel, leaving the skin pieces with a maximum 0.5 cm thickness. The skin was then kept in refrigerated conditions until being tested, which took less than 24 h after excision. For irradiation, all experimental models were individually transferred to a petri dish, further centered under a laser output. The laser was placed 8 cm away from the surface to irradiate, presenting a beam diameter of 31.9 ± 0.5 mm. For irradiation studies, an RLTMDL-808 diode laser with a wavelength of 808 nm coupled to an optical fiber with 0.22 numerical aperture (Roithner LaserTechnik GmbH, Vienna, Austria) was used. A fixed dose of 0.58 W·cm^−2^ was equally applied to all experimental models for 15 min.

The temperature of the experimental models was monitored over time using a thermal camera (Mini Bi-spectrum radiometric detector, SN-D2-F, Sunnel, Shenzhen, China) placed perpendicularly to the irradiated surface and in parallel with the laser. The camera was coupled to a portable computer for real-time monitoring of the system temperature. The camera interface allowed to pre-define regions of interest, and the software showed the maximum temperature within each one of the selected regions allowing comparing temperatures of different areas of the experimental models in the same image and time frame. The thermal camera is only able to measure the emissivity from the surface of the model and not the temperature in depth. Herein, the goal was to compare the thermal protection of the aerogels, so for each one of the experimental models, three major groups were created: (1) Only laser group—models only subjected to direct laser irradiation; (2) Pectin-based aerogel group—models in which pectin-based aerogels were placed on top to further being subjected to laser irradiation; (3) Silica-based aerogel group—models in which silica-based aerogels were placed on top to further being subjected to laser irradiation. In the test groups including aerogels, it was important to register the temperature of two different areas: a directly irradiated area; and an area where the aerogel was placed, representing the aerogel temperature during irradiation and the temperature under the aerogel structure as soon as the laser was switched OFF and the aerogel removed. In the thermal images from the groups using pectin-based aerogels, area 2 corresponded to the area where the aerogel was placed, and thus the non-irradiated area of the model, whereas area 3 corresponded to the temperature of an area directly irradiated. Contrarily, for the tests using silica-based aerogels, area 3 corresponded to a part of the aerogel, and thus the non-irradiated area of the model, whereas area 2 corresponded to the temperature of an area directly irradiated. Moreover, skin models included three additional test groups: (4) Control group—skin grafts not subjected to any kind of treatment or further manipulation/modification; (5) Pectin control group—skin grafts only placed in contact with pectin-based aerogels without any exposure to laser irradiation; and (6) Silica control group—skin grafts only placed in contact with silica-based aerogels without any exposure to laser irradiation. Test groups 5 and 6 were used to assess the topical safety of the aerogels and to rule out any possible skin irritations or further alterations caused by direct contact with the aerogels.

After monitoring possible macroscopical alterations and temperature variations over the irradiation process of the experimental models, pig and human skin models were also subjected to histopathological analysis. After irradiation, the skin grafts were preserved and fixed in 10% formalin. Later, the same samples were paraffin-embedded and cut into five-micrometer sections for hematoxylin-eosin staining (H&E staining). The prepared histology slices were analyzed in an Olympus BX51 microscope (Olympus Corporation, Tokyo, Japan), and images were taken using an Olympus U-TV1X-2 color camera.

### 4.6. Preliminary In Vivo Safety Assays Using Artemia salina Model

The safety of the aerogels was assessed using *Artemia salina* as preliminary in vivo model [53,54]. The assay started with the dissolution of commercial sea water salt in tap water according to the supplier instructions, and the subsequent hatching of *Artemia salina* eggs in that solution for 48 h at a temperature ranging from 25 to 30 °C, under aeration and continuous illumination. Prior incubation with aerogels, 1000 µL of artificial sea water containing 10–15 nauplii were transferred to individual wells from a 24-well plate. Then, the aerogel was added to the testing wells. One hundred microliters of artificial sea water and 100% of DMSO were used as negative and positive controls of mortality %, respectively. The nauplii were incubated with the testing solutions for 24 h in the same growing condition. After incubation, the dead *artemia* in each well was accounted for with the help of a magnifying glass, and 100 µL of 100% of DMSO was added to kill the remaining alive *artemia*. Twenty-four hours later, the number of total *artemia* was accounted for each well, and the mortality % was calculated according to Equation (7).

(7) $$Mortality\;\%=\frac{Dead_{24h}}{Dead_{Total}}\times 100$$

where *Dead*_24h_ is the number of dead nauplii after 24 h incubation with the samples and *Dead_Total_* is the total number of nauplii in each well. All samples were tested three times with four replicates each time.

In a posterior phase, the same assay was repeated but using pectin-based hydrogels with different pectin concentrations instead of aerogels. In this case, instead of transferring 1000 µL of artificial sea water containing 10–15 nauplii to each well from a 24-well plate, only 900 µL of artificial sea water containing the same amount of nauplii were transferred. Moreover, instead of adding small pieces of a rigid aerogel to the testing groups there were added 100 µL of each one of the pectin solutions.

### 4.7. Human Skin Compatibility Tests

The skin compatibility of the aerogels was tested in human volunteers recruited by a certified clinical trial company, PhD Trials^®^, Lda. A single-center, open-label, subject-blinded, randomized PT study was carried out on healthy subjects, with each subject being used as its own control. The methods applied were based on a modified version of the proposal from Marzulli and Maibach on Human Repeated Insult Patch Test for delayed contact hypersensitivity: HRIPT [55], and the skin compatibility was confirmed by a dermatologist. The aerogels were placed inside occlusive patches Finn Chamber^®^ standard fixed on the volunteers’ back with a hypoallergenic adhesive: Scanpor^®^ (inner diameter: 8 mm, surface: 50 mm^2^). The contact of the skin with the materials was kept for 48 ± 5 h, and the skin conditions were evaluated before patching on the first day and 15 min, 24 h, and 48 h after removal of the patch. For this study, a group of 24 subjects (12 per each aerogel type) with ages between 18 to 70 years, female and male, phototype (Fitzpatrick) I to IV, and to all types of skin was randomized into two test groups: pectin-based and silica-based aerogels. All the tests were made according to the Declaration of Helsinki and received the approval of PhD Trials^®^’ Ethics Committee (protocol code PT.02.01 approved on 27 December 2019).

### 4.8. Statistical Analysis

All results are presented as mean ± standard deviation (SD) for a specified *n*. Statistical analyses were carried out using GraphPad Prism 8^®^ (San Diego, CA, USA), and differences were considered significant when *p*-value < 0.05. Two-way ANOVA followed by Sidak’s multiple comparisons test was used to compare physicochemical properties of both aerogels as well as to compare the overall temperature increment observed after 15 min of irradiation. Two-way ANOVA followed by Tukey’s multiple comparisons test was used to compare temperature increments over time in different test groups from each experimental model. Lastly, ordinary one-way ANOVA followed by Dunnett’s multiple comparisons test was used to compare *artemia* mortality % from both aerogels.

## Figures and Tables

**Table 1:** Physicochemical properties of the aerogels prepared.

Aerogel	Volume Shrinkage %	*ρ_bulk_* (g cm^−3^)	*A_BET_* (m^2^ g^−1^)	*V_p,BJH_* (cm^3^ g^−1^)	*d_p,BJH_* (nm)
Pectin-based	83.5 ± 5.5	0.334 ± 0.092	330 ± 17	1.3 ± 0.1	10.3 ± 0.5
Silica-based	21.8 ± 2.8 ****	0.159 ± 0.009	726 ± 36 ****	2.4 ± 0.1	9.1 ± 0.5

*ρ_bulk_*: bulk density. *A_BET_*: specific surface area. *V_p,BJH_*: pore volume and *d_p,BJH_*: pore diameter obtained from BJH method. Data are represented as mean ± SD. Statistical significance is represented as **** *p* < 0.0001.

**Table 2:** Relative degree of lesion of the skin alterations observed for each one of the human skin test groups.

Test Group	Non-Irradiated	Irradiated
Only human skin	NO	++
Human skin + Pectin-based aerogel	+	+++
Human skin + Silica-based aerogel	+	+++

NO—Not observed; + mild lesion; ++ moderate lesion; +++ severe lesion.

## Data Availability

Not applicable.

## Supplementary Materials

The following supporting information can be downloaded at: https://www.mdpi.com/article/10.3390/gels8080485/s1, Figure S1: Temperature increment observed for agar phantoms throughout laser irradiation with a NIR laser. Statistical significance represents **** *p* < 0.0001 compared with all the other testing groups and # *p* < 0.05 compared with all the other groups except the group irradiated on top of a pectin aerogel: non-irradiated area (corresponds to the aerogel temperature); Figure S2: Temperature increment observed for pig skin ex vivo grafts throughout laser irradiation with a NIR laser. Statistical significance represents **** *p* < 0.0001 compared with all the other testing groups; # *p* < 0.05 compared with Only laser group, and + *p* < 0.05 compared with groups directly irradiated from both aerogels; Figure S3: Temperature increment observed for human skin ex vivo grafts throughout laser irradiation with a NIR laser. Statistical significance represents **** *p* < 0.0001 compared with all the other testing groups. Table S1: Summarized results from skin compatibility tests.
