# Supplementary material for: Biological Thermal Performance of Organic and Inorganic Aerogels as Patches for Photothermal Therapy
Source: Gels. 2022 Aug 3;8(8):485. doi: 10.3390/gels8080485 (PMC9407269; doi:10.3390/gels8080485)
Supplement: Supplementary file 1 [file gels-08-00485-s001.zip › gels-1829453-supplementary.pdf]

Supporting Information

Biological thermal performance of organic and inorganic aerogels as patches for photothermal therapy

Tânia Ferreira-Gonçalves [1,2], Ana Iglesias-Mejuto [3], Teresa Linhares [4,5], João M. P. Coelho [2], Pedro Vieira [6], Pedro Faísca [7], José Catarino [8], Pedro Pinto [1], David Ferreira [9], Hugo A. Ferreira [2], Maria Manuela Gaspar [1], Luísa Durães [4], Carlos A. García-González [3] and Catarina Pinto Reis [1,2,*]

[1] Research Institute for Medicines (iMed.ULisboa), Faculty of Pharmacy, Universidade de Lisboa, Av. Professor Gama Pinto, 1649-003 Lisboa, Portugal; taniag1@edu.ulisboa.pt (T.F.-G.); pfcpinto@ff.ulisboa.pt (P.P.); mgaspar@ff.ulisboa.pt(M.M.G.)

[2] Instituto de Biofísica e Engenharia Biomédica, Faculdade de Ciências, Universidade de Lisboa, Campo Grande, 1749-016 Lisboa, Portugal; jmcoelho@fc.ul.pt (J.M.P.C.); hugoferreira@campus.ul.pt (H.A.F.)

[3] I+D Farma Group (GI-1645), Department of Pharmacology, Pharmacy and Pharmaceutical Technology, Faculty of Pharmacy, iMATUS and Health Research Institute of Santiago de Compostela (IDIS), Universidade de Santiago de Compostela, E-15782 Santiago de Compostela, Spain; ana.iglesias.mejuto@rai.usc.es (A.I.-M.); carlos.garcia@usc.es (C.A.G.-G.)

[4] University of Coimbra, CIEPQPF, Department of Chemical Engineering, 3030-790 Coimbra, Portugal; tcalin-hares@gmail.com (T.L.); luisa@eq.uc.pt (L.D.)

[5] 2C2T-Centre for Textile Science and Technology, University of Minho, Campus of Azurém, 4800-058, Guimarães, Portugal.

[6] Physics Department, NOVA School of Science and Technology (Campus de Caparica), 2829-516 Caparica, Portugal; pmv@fct.unl.pt (P.V.)

[7] CBIOS-Research Center for Biosciences & Health Technologies, Universidade Lusófona de Humanidades e Tecnologias, Campo Grande 376, 1749-024 Lisboa, Portugal; pedrofaisca@ulusofona.pt (P.F.)

[8] Faculdade de Medicina Veterinária, Universidade Lusófona de Humanidades e Tecnologias, Campo Grande 376, 1749-024 Lisboa, Portugal; p5663@ulusofona.pt (J.C.)

[9] Comprehensive Health Research Centre (CHRC), Departamento de Desporto e Saúde, Escola de Saúde e Desenvolvimento Humano, Universidade de Évora, Largo dos Colegiais, 7004-516 Évora, Portugal; david.ferreira@uevora.pt (D.F.)

[*] Correspondence: catarinareis@ff.ulisboa.pt; Tel.: +351-217-946-429 (ext. 14244); Fax: +351-217-946-470

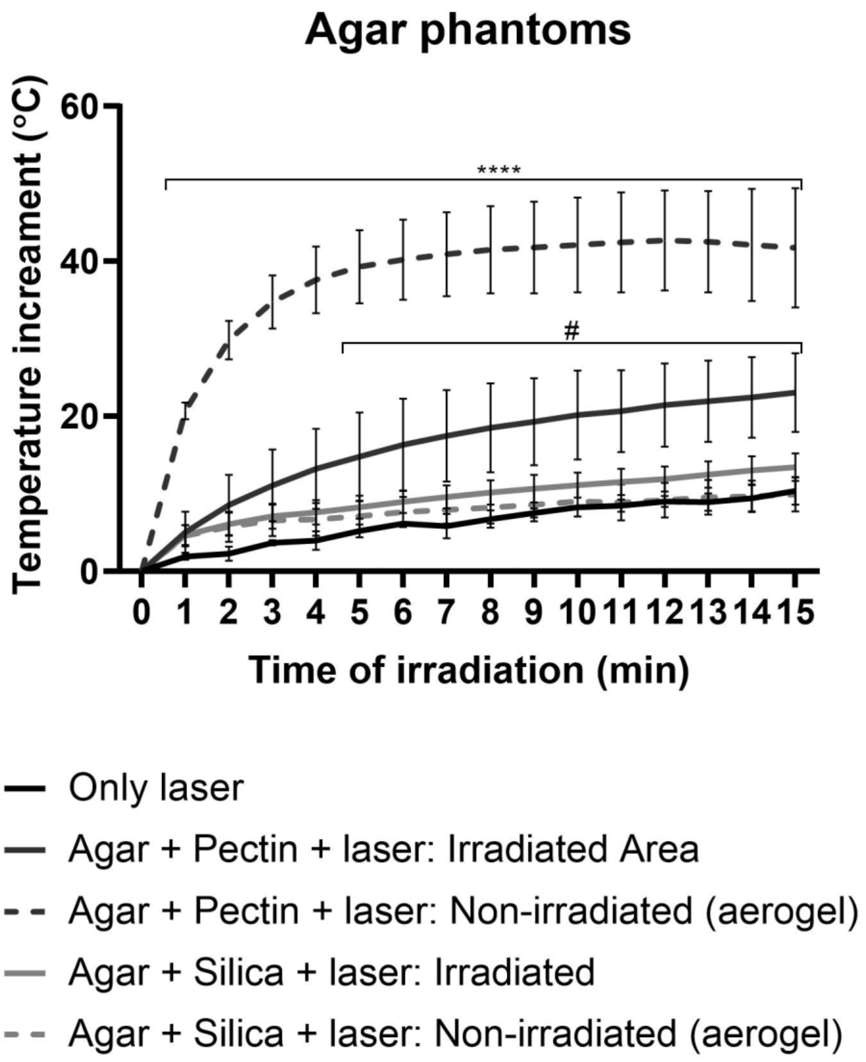

Figure S1: Temperature increment observed for agar phantoms throughout laser irradiation with a NIR laser. Statistical significance represents **** $p < 0.0001$ compared with all the other testing groups and # $p < 0.05$ compared with all the other groups except the group irradiated on top of a pectin aerogel: non-irradiated area (corresponds to the aerogel temperature).

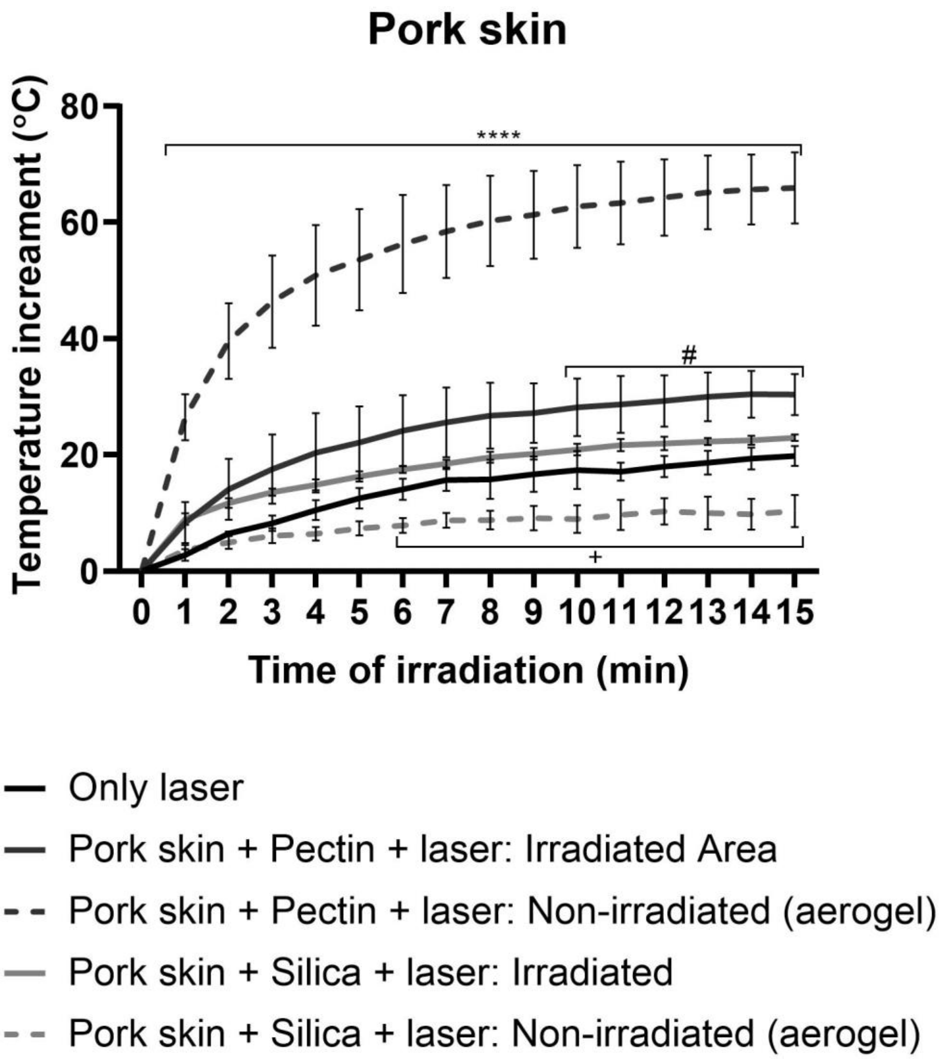

Figure S2: Temperature increment observed for pig skin ex vivo grafts throughout laser irradiation with a NIR laser. Statistical significance represents **** $p < 0.0001$ compared with all the other testing groups; # $p < 0.05$ compared with Only laser group, and + $p < 0.05$ compared with groups directly irradiated from both aerogels.

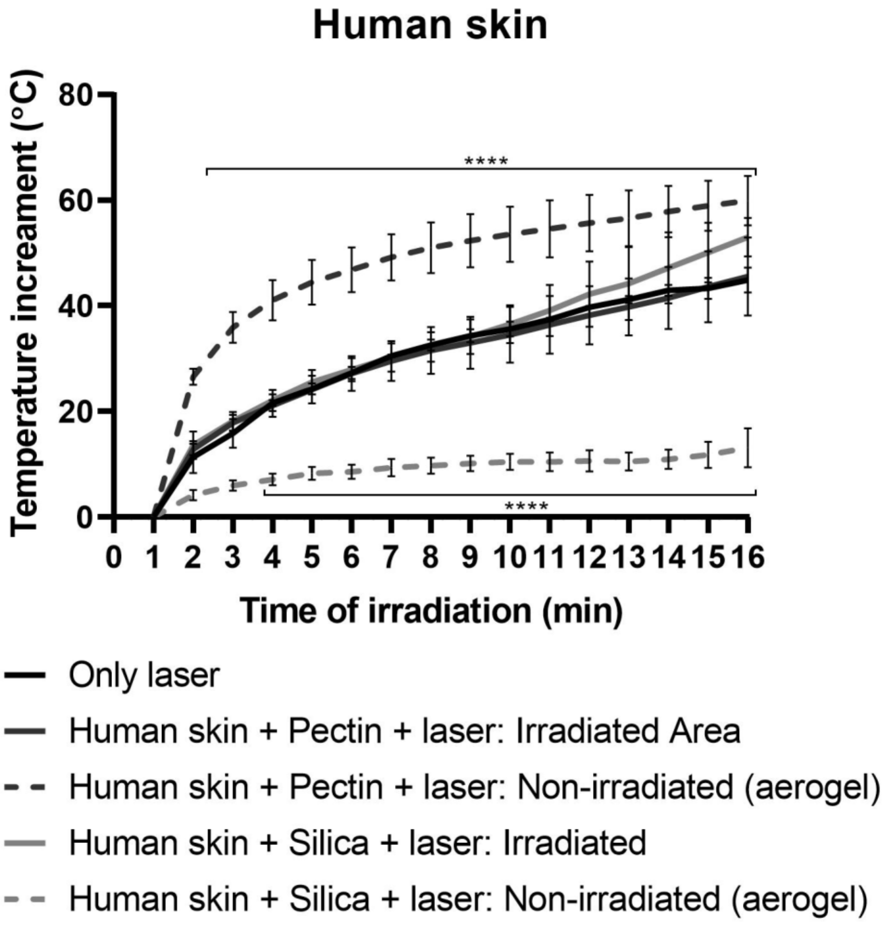

Figure S3: Temperature increment observed for human skin ex vivo grafts throughout laser irradiation with a NIR laser. Statistical significance represents **** $p < 0.0001$ compared with all the other testing groups.

Table S1. Summarized results from skin compatibility tests.

Aerogel Material	Control time after patch removal	Type of reaction[1]	Mean daily irritation score (Mdis)[2]	% of reactive subjects
**Pectin-based aerogel**	15 min	None	0	0
	24 h	None	0	0
	48 h	None	0	0
**Silica-based aerogel**	15 min	None	0	0
	24 h	None	0	0
	48 h	None	0	0

[1] A dermatologist evaluated the skin conditions looking for any visible signs of reactivity such as erythema, oedema, vesicle, bulla, papule or scab. [2] Individual daily irritation score is primarily calculated for each individual (I_{dis}) based on the sum of marks attributed to visible signs depending on their intensity: erythema, oedema, vesicle, papule (from 1 to 2 or 3) and bulla, scab (2 if present). Then, the mean daily irritation score (M_{dis}) was calculated according Mdis = $\sum$ (I_{dis}) / number of subjects.
